# Pectate Lyase Genes Abundantly Expressed During the Infection Regulate Morphological Development of *Colletotrichum camelliae* and *CcPEL16* Is Required for Full Virulence to Tea Plants

**DOI:** 10.1128/msphere.00677-22

**Published:** 2023-01-24

**Authors:** Hong Jiang, Qinghai Cao, Xinchao Wang, Wuyun Lv, Yuchun Wang

**Affiliations:** a College of Tea Science and Tea Culture, Zhejiang A&F University, Hangzhou, Zhejiang, China; b Tea Research Institute, Chinese Academy of Agricultural Sciences/National Center for Tea Improvement/Key Laboratory of Tea Biology and Resources Utilization, Ministry of Agriculture and Rural Affairs of the People's Republic of China, Hangzhou, Zhejiang, China; University of Georgia

**Keywords:** pectate lyase genes, *Colletotrichum camelliae*, tea plant, secretory protein, pathogenicity

## Abstract

Colletotrichum camelliae is the dominant species causing foliar diseases of tea plants (Camellia sinensis) in China. Transcriptome data and reverse transcription-quantitative PCR (qRT-PCR) analysis have demonstrated that the pectate lyase genes in *C. camelliae* (*CcPEL*s) were significantly upregulated during infectious development on tea plants (cv. *Longjing43*). To further evaluate the biological functions of *CcPEL*s, we established a polyethylene glycol (PEG)-mediated protoplast transformation system of *C. camelliae* and generated targeted deletion mutants of seven *CcPEL*s. Phenotypic assays showed that the genes contribute to mycelial growth, conidiation, and appressorium development. The polypeptides encoded by each *CcPEL* gene contained a predicted N-terminal signal peptide, and a yeast invertase secretion assay suggested that each CcPEL protein could be secreted. Cell death-suppressive activity assays confirmed that all seven CcPELs did not suppress Bax-induced cell death in tobacco leaf cells. However, deletion of *CcPEL16* significantly reduced necrotic lesions on tea leaves. Taken together, these results indicated that *CcPEL*s play essential roles in regulating morphological development, and *CcPEL16* is required for full virulence in *C. camelliae*.

**IMPORTANCE** In this study, we first established a PEG-mediated protoplast transformation system of *C. camelliae* and used it to investigate the biological functions of seven pectate lyase genes (*CcPEL*s) which were abundantly expressed during infection. The results provided insights into the contributions of pectate lyase to mycelial growth, conidial production, appressorium formation, and the pathogenicity of *C. camelliae.* We also confirmed the secretory function of CcPEL proteins and their role in suppressing Bax-induced cell death. Overall, this study provides an effective method for generating gene-deletion transformants in *C. camelliae* and broadens our understanding of pectate lyase in regulating morphological development and pathogenicity.

## INTRODUCTION

The tea plant Camellia sinensis (L.) O. Kuntze has been widely cultivated as an important economic crop in more than 60 countries all over the world (1). Foliar diseases caused by Colletotrichum camelliae, a *C. gloeosporioides* complex species, affect tea plant cultivation and the yield and quality of tea (2–4). As a dominant fungal pathogen occuring on *Ca. sinensis*, *C. camelliae* causes anthracnose in tea leaves (3, 5). *Colletotrichum* always invades *Ca. sinensis* from the back of the young leaves and incubates for 1 to 2 weeks, making it difficult to detect the disease and control it in a timely manner (6).

*Colletotrichum* spp. possess unique a intracellular hemi-biotrophic lifestyle and serve as a model pathogen for molecular biological and genetic studies (7). Most *Colletotrichum* species, such as *C. fructicola*, *C. lagenarium*, *C. lindemuthianum*, *C. gloeosporioides*, *C. graminicola*, and *C. truncatum*, initially establish infection through a brief biotrophic phase (8–13). They differentiate melanized appressoria which are separated from the germ tubes of germinated conidia (14). During infection, the appressoria penetrate the host cuticle and cell wall through a combination of enormous turgor pressure and enzymatic degradation (15). At this early stage of infection, the pathogen secretes small proteinaceous and nonproteinaceous molecules, known as effectors, into the host to manipulate the host cell physiological metabolism and establish biotrophy (12, 16). Phytopathogens deploy effectors to facilitate their own infection as virulence factors and deploy toxins or triggering host defense responses as avirulence factors and elicitors (17). For *C. camelliae*, conidia can germinate and form melanized dome-shaped appressoria and then develop infectious hypha inside tea leaf cells during the biotrophic stage (3). In the process of invading tea plants, *C. camelliae* probably secretes effectors into the cytoplasm of *Ca. sinensis* to facilitate infection, which triggers an effector-triggered immune response, inducing a hypersensitive response and cell death (3, 18). Based on transcriptome data, pectate lyase genes in *C. camelliae* (*CcPEL*s) as the candidate effectors showed extremely high expression during this phase of infection, which suggests that *CcPEL*s play critical roles in regulating the pathogenesis of *C. camelliae* to tea plants.

Pectate lyases (PELs), as plant cell wall-degrading enzymes (PCWDEs), serve as virulence factors in pathogens and play an essential role during the infection process (19). Pectate lyases cleave pectate via a β-elimination reaction, macerating and disassembling the plant cell wall and tissues (20). In several plant-pathogenic fungi, PELs have been shown to be involved in enhancing pathogenicity and inducing plant immune responses. In Fusarium solani f. sp. *pisi*, both *pelA* and *pelB* were expressed when infecting pea plants (Pisum sativum) (21). In the avocado pathogen *C. gloeosporioides*, pectate lyase B was an important virulence factor and could induce host defense mechanisms (22). PEL, encoded by *pecCl1*, is an important determinant for the aggressiveness of *C. lindemuthianum* against the common bean (23)*. CcPELA* gene-disrupted mutants of *C. coccodes* showed reduced virulence toward tomato fruits (24). In *Verticillium dahlia*, a pectate lyase (VdPEL1) contributed to virulence and induced plant defense responses (19). In another study, 22 *PEL* genes in Phytophthora capsici were cloned, and the combined silencing of *PcPL1*, *PcPL15*, *PcPL16*, and *PcPL20* severely affected virulence on pepper (25). Gene deletion and constitutive expression of pectate lyase gene 1 resulted in diminished virulence of Magnaporthe oryzae (26). However, the biological functions of *CcPEL*s in *C. camelliae* have not been reported.

In this study, seven *CcPELs* were knocked-out via an initially established polyethylene glycol (PEG)-mediated protoplast transformation system. Targeted deletion of *CcPEL2* resulted in pleiotropic defects in aerial hyphal growth and conidiation. The Δ*CcPEL6* mutants showed defects in vegetative growth and appressorium formation, and Δ*CcPEL25* mutants displayed reduced vegetative growth and decreased conidiation. In addition, the Δ*CcPEL16* and Δ*CcPEL26* mutants exhibited sparse aerial mycelia on PDA plates. Targeted deletion of *CcPEL26* resulted in defects in conidiation and appressorium formation. These results indicated that pectate lyase is involved in regulating morphogenesis in *C. camelliae*. Signal peptide activity analysis showed that all seven CcPELs could be secreted. Pathogenicity tests demonstrated that *CcPEL16* plays indispensable roles in virulence toward tea plant leaves. Considering these results, we conclude that pectate lyase plays important roles in vegetative growth, asexual sporulation, appressorium development, and pathogenicity in *C. camelliae*.

## RESULTS

### The expression pattern of *CcPEL*s in different stages of infection.

**FIG 1:**
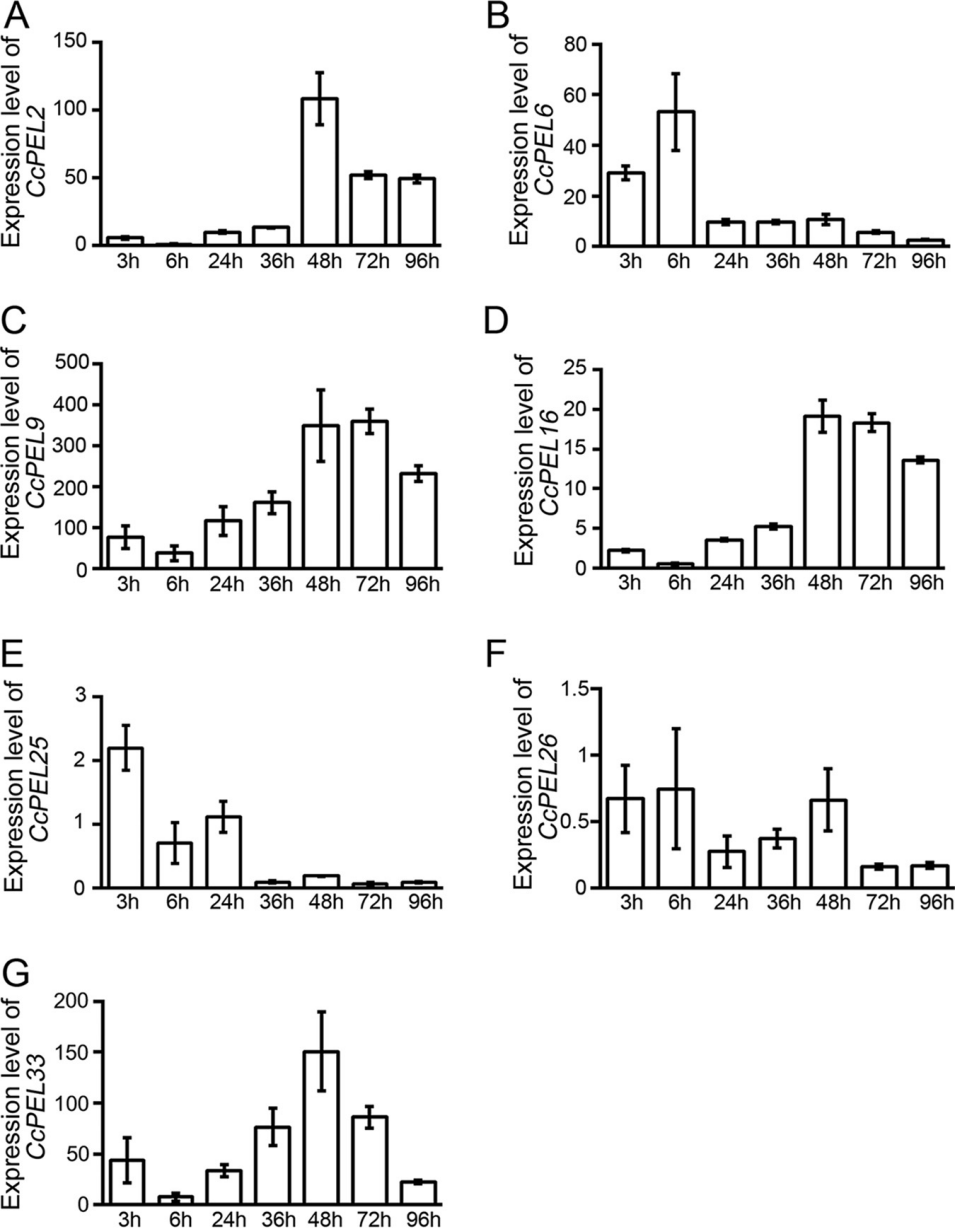
Expression pattern of *CcPEL*s during different stages of Colletotrichum camelliae infection. Transcriptional expression levels of *CcPEL2* (A), *CcPEL6* (B), *CcPEL9* (C), *CcPEL16* (D), *CcPEL25* (E), *CcPEL26* (F), and *CcPEL33* (G) were investigated by reverse transcription-quantitative PCR (qRT-PCR) analysis. The *CcNEW1* gene was used as the internal reference. Error bars represent standard deviation.

Based on transcriptome data, the *CcPEL*s as the candidate effectors were significantly upregulated during the infection (3). As a result, we performed reverse transcription-quantitative PCR (qRT-PCR) assays to further confirm their expression patterns. Total RNA was extracted from tea leaves at 3, 6, 24, 36, 48, 72, and 96 h after inoculation with a highly virulent isolate of *C. camelliae* (LS_19) (27). qRT-PCR analyses showed that expression of *CcPEL*s, including *CcPEL2*, *CcPEL6*, *CcPEL9*, *CcPEL16*, *CcPEL25*, *CcPEL26*, and *CcPEL33*, was induced and significantly upregulated during infection (Fig. 1). The expression patterns of *CcPEL2*, *CcPEL9*, *CcPEL16* and *CcPEL33* were comparable, showing considerable transcript accumulation with peaks at 48 or 72 hpi (hours postinoculation). In addition, with the development of infection, the general expression levels of the four genes gradually increased (Fig. 1). In contrast, *CcPEL6* and *CcPEL25* showed high expression at the early stage of infection (3 or 6 hpi), and expression levels significantly decreased in the later stage. The expression pattern of *CcPEL26* was different from that of the other six genes, exhibiting a slight transcript accumulation at different stages of infection (Fig. 1). Notably, compared with those of the other genes, the overall expression levels of *CcPEL25* and *CcPEL26* were lower at each stage of infection. These results suggest that the seven *CcPELs* probably play important roles in the process of *C. camelliae* infecting tea plants.

### Establishment of PEG-mediated protoplast transformation system of *C. camelliae*.

**FIG 2:**
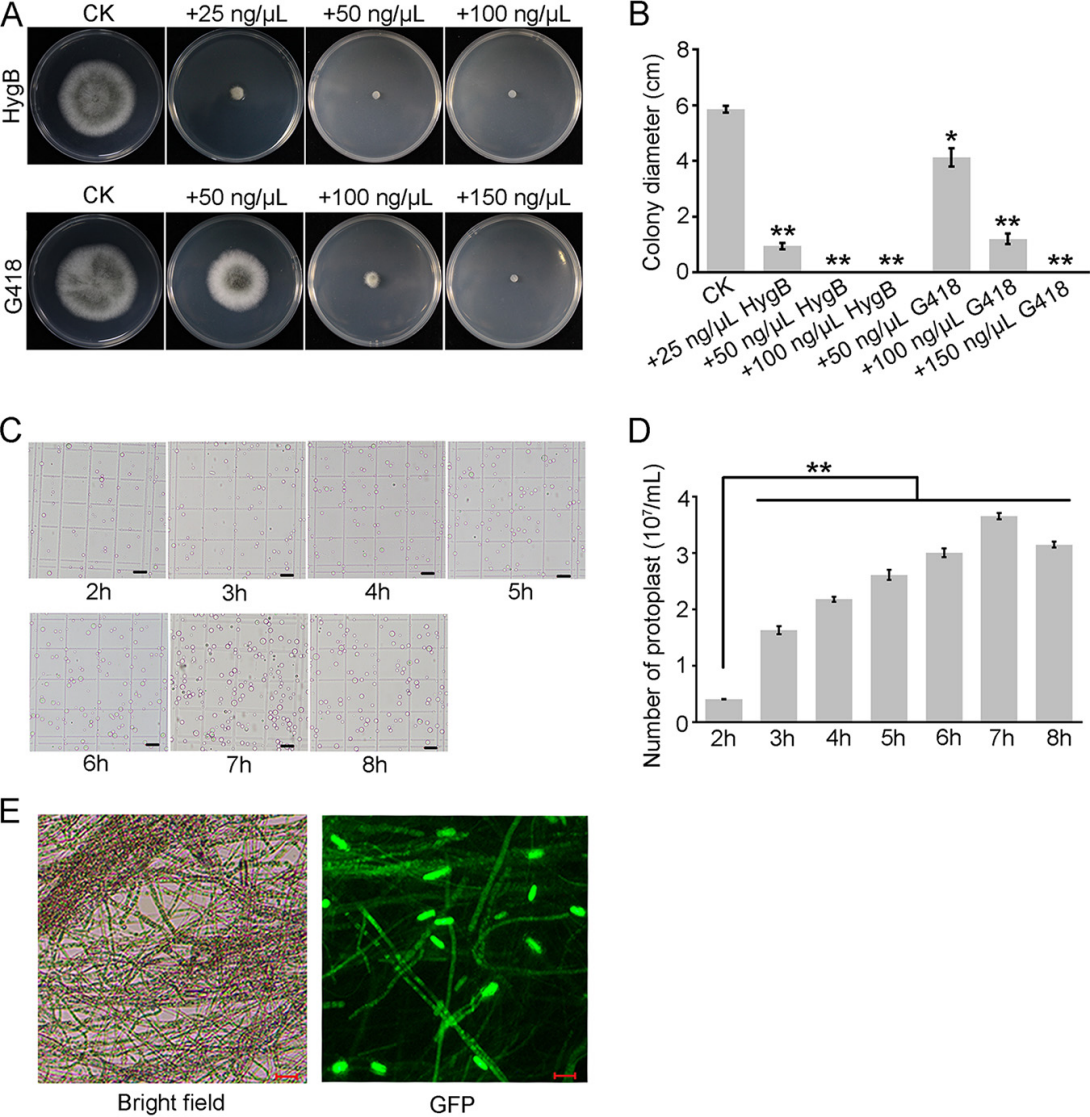
Optimal parameters used for polyethylene glycol (PEG)-mediated protoplast transformation in *C. camelliae*. (A) Colony growth of the wild-type strain LS_19 on potato dextrose agar (PDA) with different concentrations of hygromycin or G418. LS_19 was incubated at 28°C for 4 days. (B) Statistical analysis of inhibition rate of radial growth of LS_19 strain. Error bars represent standard deviation. *, *P* < 0.05; **, *P* < 0.01. (C) Observation of protoplasts under light microscopy. Scale bar = 20 μm. (D) Statistical analysis of protoplast yields at different reaction times. Error bars represent standard deviation. **, *P* < 0.01. (E) Fluorescence microscopy analysis for detection of green fluorescent protein (GFP) signal in *C. camelliae* transformants bearing pBluescript KS II (+)-GFP. Scale bar = 20 μm.

To investigate the biological functions of *CcPELs* in *C. camelliae*, it is necessary to generate targeted gene deletion mutants of the *CcPEL*s. The PEG-mediated protoplast transformation system has been widely used for genetic transformation in plant-pathogenic fungi (28–30). Thus, we attempted to establish a PEG-mediated protoplast transformation system for generating deletion mutants in *C. camelliae*. The *C. camelliae* strain LS_19 was used as the wild-type strain. Fifty μg/mL hygromycin B (HygB) or 100 μg/mL G418 completely inhibited mycelial growth of LS_19, suggesting its extreme sensitivity to hygromycin B and G418 (Fig. 2A and B). Therefore, 50 μg/mL HygB or 100 μg/mL G418 was used for transformant selection. Protoplast yield was related to the incubation time in the mixed lytic enzyme generation, which was low, 0.41 ± 0.01 × 10^7^ protoplasts/mL at 2 hpi, and increased with the extension of incubation time. At 7 hpi, the yield was the highest, up to 3.66 ± 0.06 × 10^7^ protoplasts/mL (Fig. 2C). To verify the efficiency of the PEG-mediated protoplast transformation system, a vector carrying green fluorescent protein (GFP) tag was transformed into a protoplast of the LS_19 strain. The resulting transformants were confirmed by fluorescence microscopy: GFP fluorescence could be observed in the hyphal cells of nearly 50% transformants (Fig. 2D). The results proved that the high-efficiency PEG-mediated protoplast transformation system can be used for genetic transformation and generating transformants lacking the target genes.

### Targeted gene geletion of *CcPEL*s in *C. camelliae*.

We generated targeted gene deletion mutants of seven *CcPEL*s via a homologous recombination strategy with the PEG-mediated protoplast transformation system. The entire open reading frame of each *CcPEL* in the wild-type strain LS_19 was replaced, respectively, by a HygB resistance cassette. The gene replacement events in the null mutants were confirmed by PCR analysis using two primer pairs (Fig. S1; Table S1).

10.1128/msphere.00677-22.1 FIG S1 Targeted gene replacement of *CcPEL*s and confirmation by PCR analysis. (A) Construction of fused fragments used for the vectors pKOV21-CcPEL and targeted replacement of *CcPEL*s. (B) PCR analysis to confirm the existence of *CcPEL* genes and the size of *CcPEL*s or *HPH* fragments. Download FIG S1, JPG file, 1.4 MB. Copyright © 2023 Jiang et al. 2023 Jiang et al. https://creativecommons.org/licenses/by/4.0/ This content is distributed under the terms of the Creative Commons Attribution 4.0 International license.

10.1128/msphere.00677-22.2 TABLE S1 Primers used in this study. Download Table S1, DOCX file, 0.02 MB. Copyright © 2023 Jiang et al. 2023 Jiang et al. https://creativecommons.org/licenses/by/4.0/ This content is distributed under the terms of the Creative Commons Attribution 4.0 International license.

### Pectate lyase is required for proper vegetative growth in *C. camelliae*.

**FIG 3:**
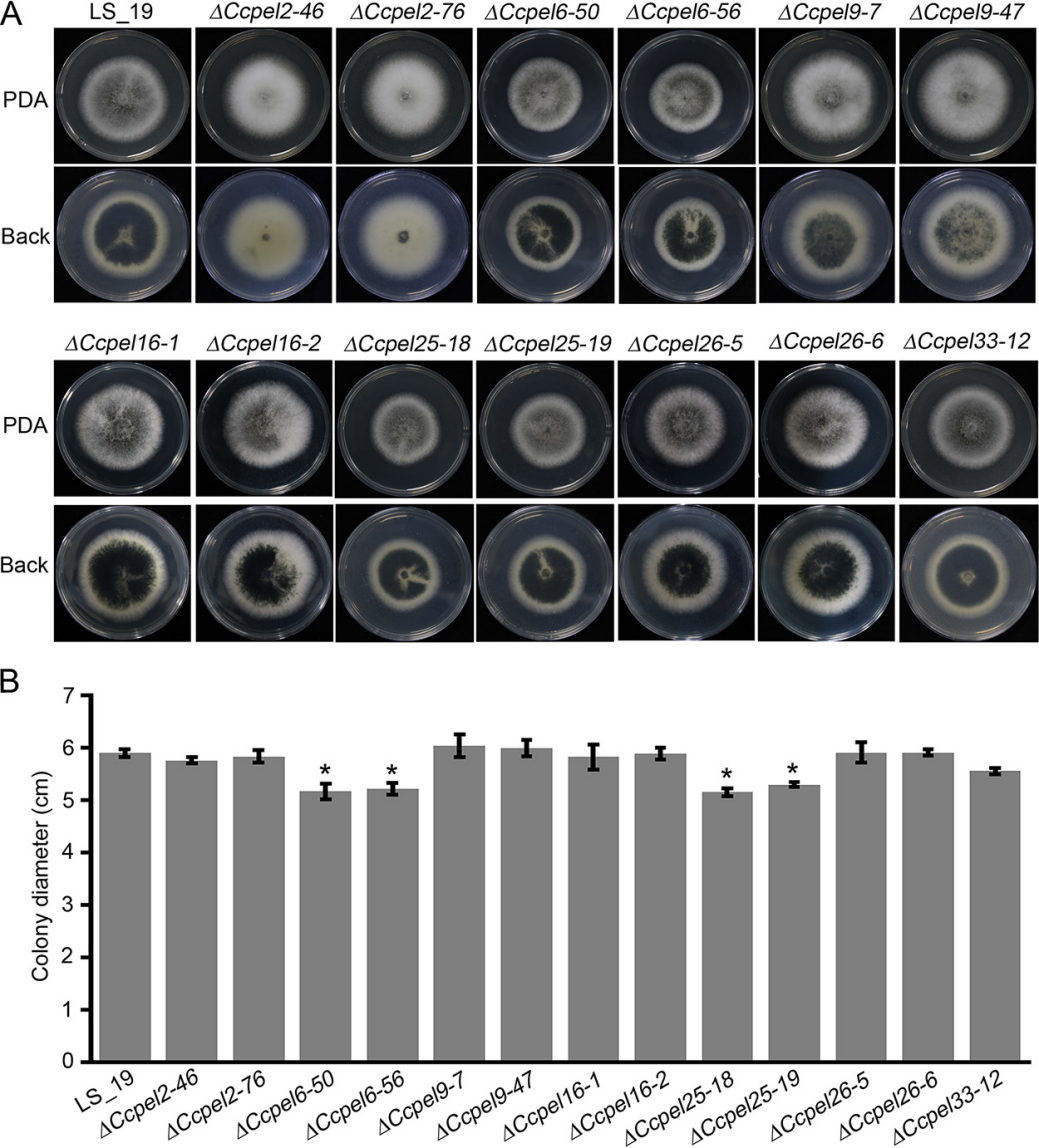
Defects of *CcPEL* mutants in hyphal growth. (A) Colony morphology of each strain cultured on PDA plates at 28°C for 4 days. (B) Radial growth of each strain on PDA plates. Error bars represent standard deviation. *, *P* < 0.05.

To evaluate the roles of all mutants of *CcPEL*s in the mycelial growth and development of *C. camelliae*, the null mutants were cultured on potato dextrose agar (PDA) plates at 28°C for 4 days. The results showed that the Δ*Ccpel33-12* mutant displayed similar colony morphology to the wild-type strain LS_19 (Fig. 3). However, compared to LS_19, the Δ*CcPEL2* and Δ*CcPEL9* mutants displayed whiter aerial hypha and produced much less pigment on the back of PDA plates (Fig. 3A). The Δ*CcPEL16* and Δ*CcPEL26* mutants exhibited sparse aerial mycelium on PDA plates, in contrast to the dense aerial mycelium observed in LS_19 (Fig. 3A). In addition, the Δ*CcPEL6-50*, Δ*CcPEL6-56*, Δ*CcPEL25-18*, and Δ*CcPEL25-19* mutants displayed a statistically significant decrease in hyphal growth, forming colonies with diameters of 5.33 (±0.31), 5.23 (±0.13), 5.15 (±0.07), and 5.38 (±0.10 cm), respectively, compared to the 5.81 (±0.17)-cm colony diameter of LS_19 (Fig. 3B). These results suggest that pectate lyase plays a role in vegetative growth in *C. camelliae*.

### Pectate lyase is involved in conidiogenesis and conidial morphology in *C. camelliae*.

**FIG 4:**
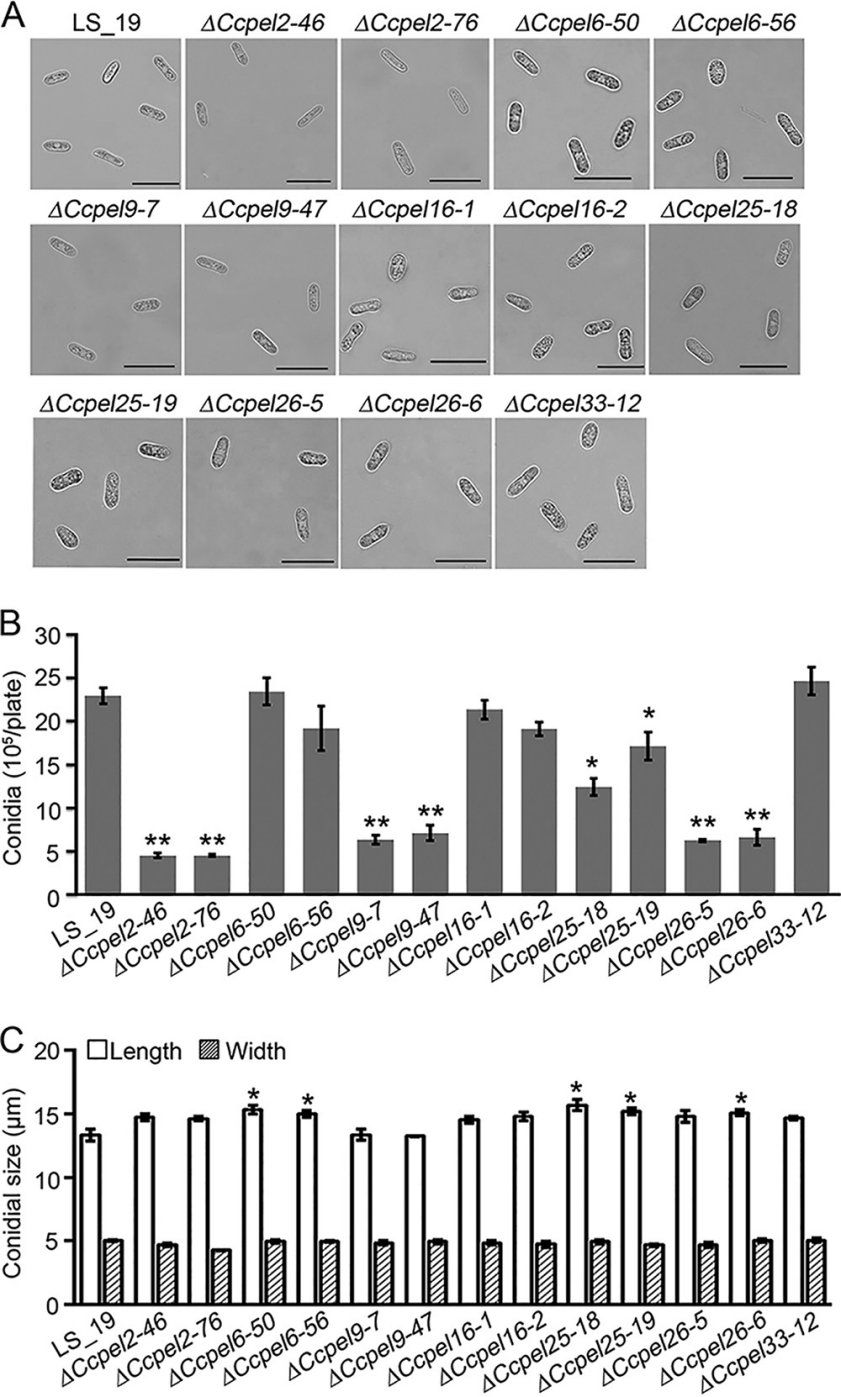
Deletion of *CcPEL*s reduces conidiation in *C. camelliae*. (A) Microscopic observation of conidia morphology. Conidia of each strain were collected from PDA plates at 28°C for 7 days. Scale bar = 20 μm. (B) Bar chart showing statistical analysis of conidiation. Error bars represent standard deviation. *, *P* < 0.05; **, *P* < 0.01. (C) Bar chart showing the length and width of conidia from each strain. Error bars represent standard deviation. *, *P* < 0.05.

The conidiation abilities of all mutants were determined according to the method described by He et al. (31). Compared with the wild-type strain LS_19, which produced 22.93 (±0.94) × 10^5^ conidia per plate, the Δ*CcPEL2-46* and Δ*CcPEL2-76* mutants, Δ*CcPEL9-7* and Δ*CcPEL9-47* mutants, Δ*CcPEL25-18* and Δ*CcPEL25-19* mutants, and Δ*CcPEL26-5* and Δ*CcPEL26-6* mutants produced significantly fewer conidia, producing 4.50 (±0.25) × 10^5^ and 4.50 (±0.15) × 10^5^ conidia, 6.33 (±0.52) × 10^5^ and 7.10 (±0.87) × 10^5^ conidia, 12.43 (±0.99) × 10^5^ and 17.1 (±1.61) × 10^5^ conidia, and 6.23 (±0.15) × 10^5^ and 6.60 (±0.95) × 10^5^ conidia, respectively, per plate (Fig. 4A). We also examined the morphology of conidia produced by each strain under a light microscope. Most conidia produced by LS_19 were cylindrical with obtuse ends and smooth-walled. Comparatively, some conidia produced by the Δ*CcPEL16-1* and Δ*CcPEL16-2* mutants narrowed at the center or toward the base (Fig. 4B). The conidial size of LS_19 was 13.31 (±0.45) × 4.98 (±0.07) μm. In contrast, the Δ*CcPEL6-50* and Δ*CcPEL6-56* mutants, Δ*CcPEL25-18* and Δ*CcPEL25-19* mutants, and Δ*CcPEL26-6* mutants produced significantly longer conidia: 15.3 (±0.35), 14.99 (±0.25), 15.7 (±0.46), 15.2 (±0.47), and 15.09 (±0.23) μm, respectively (Fig. 4C). These results indicate that pectate lyase is involved in the conidiogenesis and conidial morphology of *C. camelliae*.

### Pectate lyase contributes to appressorium development.

**FIG 5:**
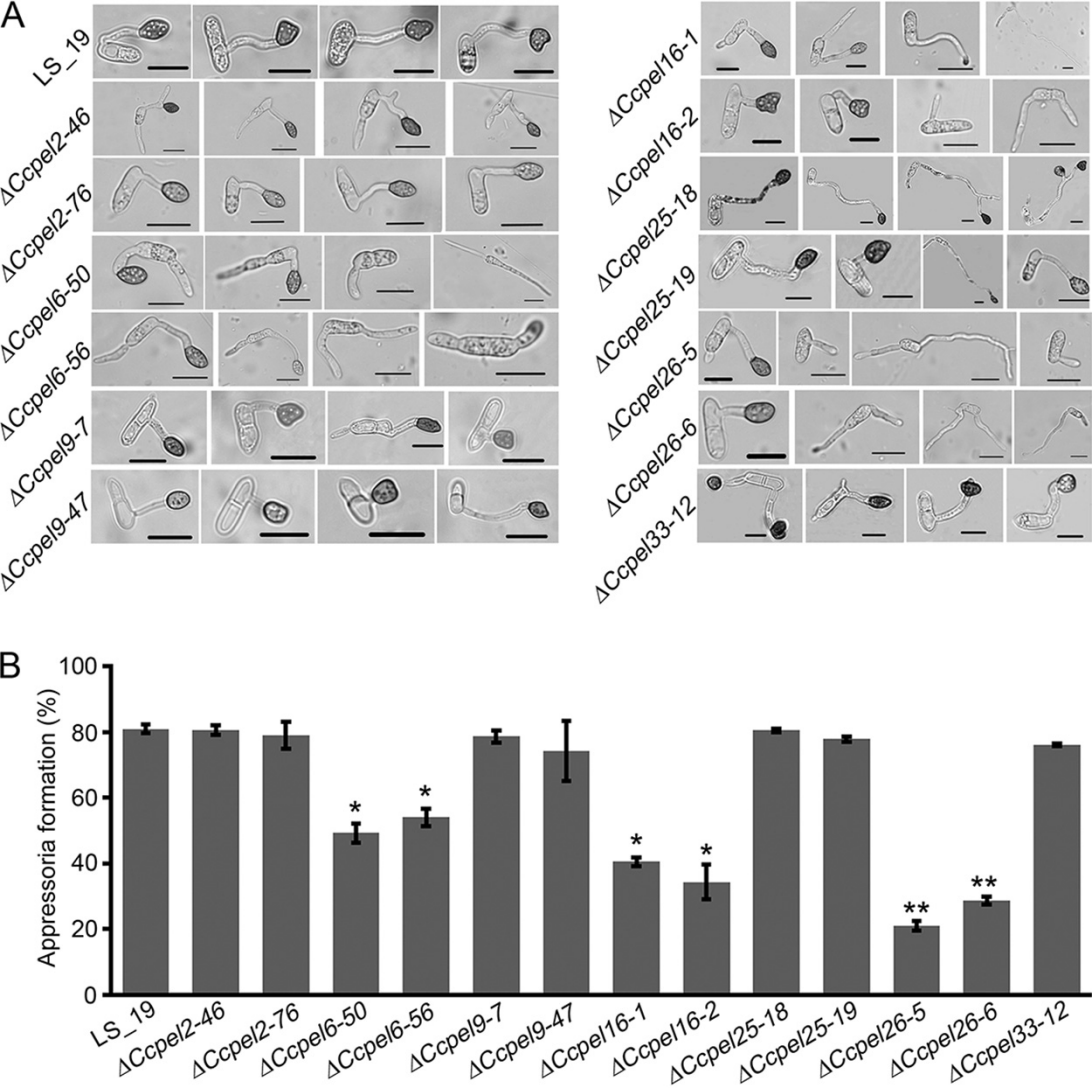
*CcPELs* are involved in appressorium formation in *C. camelliae*. (A) Microscopic observation of appressorium development of each strain. Conidia were harvested from PDA plates and allowed to form appressoria on hydrophobic surfaces in the dark (24 h). Scale bar = 20 μm. (B) Statistical analysis of appressorium formation rate. Error bars represent standard deviation. *, *P* < 0.05; **, *P* < 0.01.

To investigate the role of *CcPEL*s in appressorium development, conidia of each strain were allowed to germinate on hydrophobic coverslips. By 24 hpi, about 81% of conidia produced by the wild-type strain LS_19 germinated and formed melanized appressoria with various shapes at the tips of germ tubes. However, targeted deletion of *CcPEL6*, *CcPEL16*, and *CcPEL26* resulted in decreased appressorium formation. For the Δ*CcPEL6-50* and Δ*CcPEL6-56* mutants, only about 49% and 54% of conidia, respectively produced appressoria on hydrophobic surface. About 40% and 34% of conidia from the Δ*CcPEL16-1* and Δ*CcPEL16-2* mutants, respectively, could form appressoria. Additionally, the rate of appressorium formation in the Δ*CcPEL26-5* and Δ*CcPEL26-6* mutants significantly declined, being only around 21% and 29%, respectively (Fig. 5A and B). These results suggest that *CcPEL6*, *CcPEL16*, and *CcPEL26* are crucial for appressorium formation. In addition, the appressoria produced by the Δ*CcPEL6* and Δ*CcPEL25* mutants were more melanized compared to those produced by LS_19 (Fig. 5A). Conidia of the Δ*CcPEL25-18* mutant could produce two appressoria at one germ tube, and conidia from the Δ*CcPEL33-12* mutant germinated from cells at both ends and formed appressoria at the tips of two germ tubes (Fig. 5A). These results demonstrated that pectate lyase contributes to appressorium development in *C. camelliae*.

### Validation of the secretory function of CcPEL proteins.

**FIG 6:**
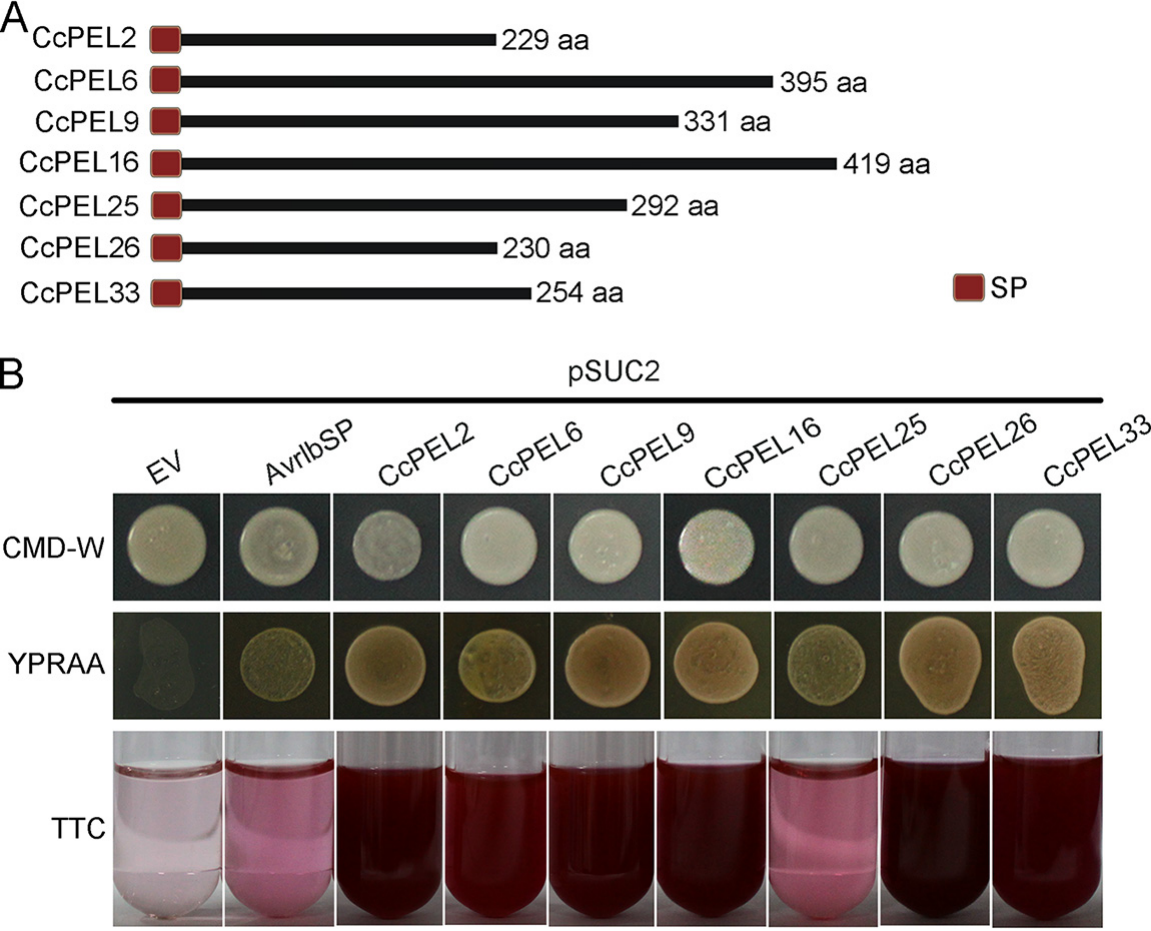
Secretion function analysis of the putative signal peptide (SP) of CcPEL proteins. (A) Putative signal peptide of each CcPEL protein. SP, signal peptide. (B) Invertase secretion analysis and 2,3,5-triphenyltetrazolium chloride (TTC) color reaction showed that the SP of each CcPEL protein could be secreted. The putative SP was fused in-frame to the invertase gene in the pSUC2 vector and transformed into yeast strain YTK12. The transformed YTK12 strains were grown on CMD-W and YPRAA media, and TTC reverted to insoluble red-colored 1,3,5-triphenylformazan (TPF). The empty vector (EV) pSUC2 was used as the negative control. The SP of Avr1b from *Phytophthora sojae* was used as the positive control.

SignalP version 5.0 (https://services.healthtech.dtu.dk/service.php?SignalP-5.0) predicted that all seven CcPELs possess a putative signal peptide (SP) at the N terminus (Fig. 6A). We performed yeast invertase secretion assays to validate the secretory function of the putative SPs via the yeast strain YTK12 and pSUC2 vector. Strain YTK12 is invertase-deficient, and pSUC2 vector contains an invertase gene but lacks methionine and a SP sequence (32). The predicted sequences of each SP were cloned into the vector pSUC2, and then transformed into cells of the yeast YTK12 mutant strain. Compared with the positive control carrying pSUC2-Avr1bSP vector, the predicted SP of CcPELs mediated the complementation of YTK12 strain growing on YPRAA medium (Fig. 6B). In addition, the invertase enzymatic activity could be further detected by the reduction of 2,3,5-triphenyltetrazolium chloride (TTC) to insoluble red-colored 1,3,5-triphenylformazan (TPF). Culture filtrates from each YTK12 strain carrying pSUC2-CcPELs exhibited a red color with TTC treatment. In contrast, the YTK12 strain carrying pSUC2 empty vector, as the negative control, was not able to grow on YPRAA medium, and its culture filtrates remained colorless after TTC treatment (Fig. 6B). These results suggested that the CcPEL proteins carry a functional secretory SP.

### Seven *CcPEL*s cannot suppress Bax-induced cell death in *Nicotiana tabacum*.

**FIG 7:**
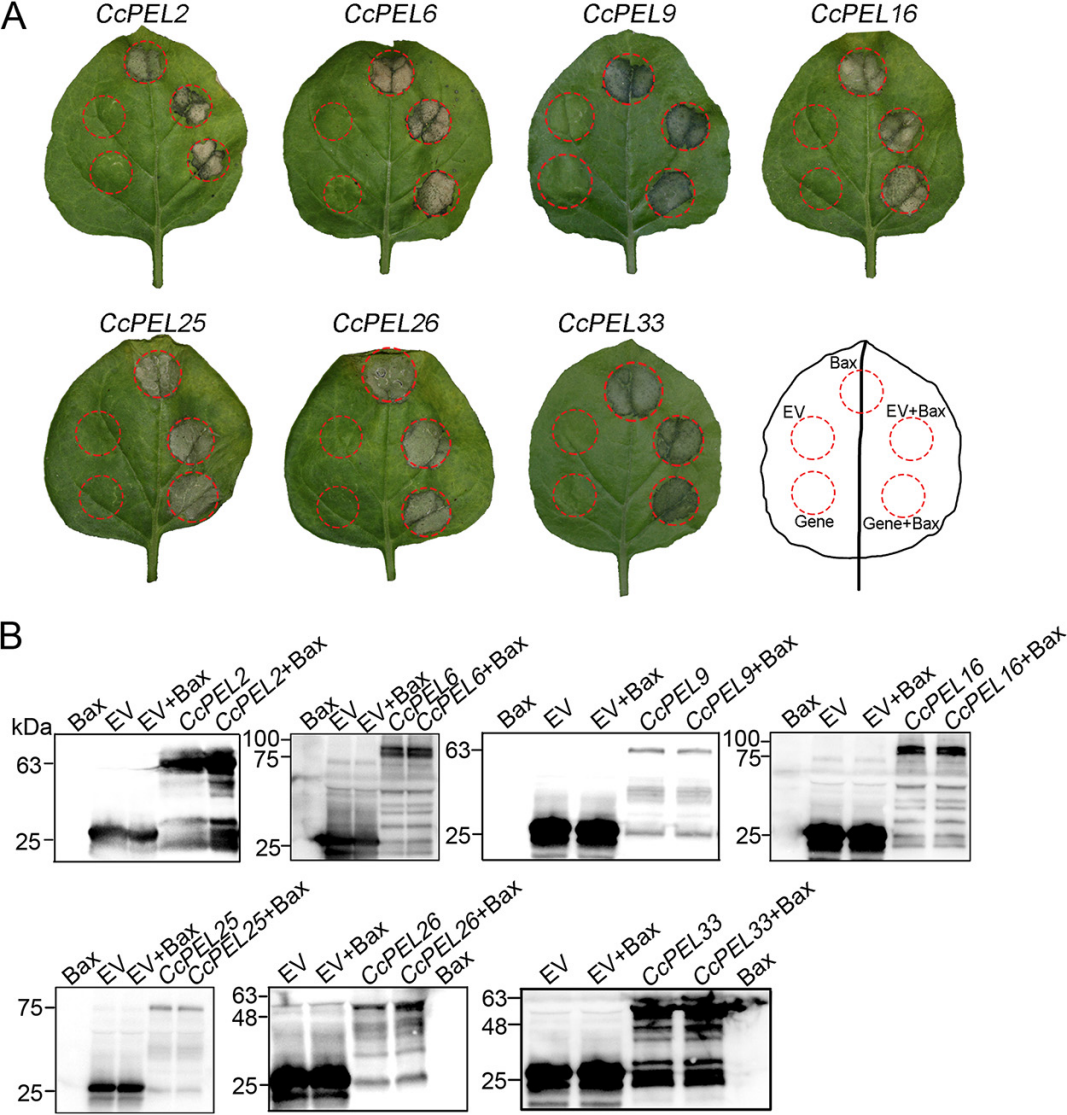
CcPEL proteins without signal peptide could not suppress Bax-induced cell death. (A) Leaves of Nicotiana tabacum were inoculated with Agrobacterium tumefaciens carrying the pGR107-GFP vector with the *BAX* gene or *CcPEL*s without signal peptide sequences. Leaves were inoculated with the empty vector (EV) pGR107-GFP as a control. Red circle indicated infiltrated regions. Photographs were taken 7 days after infiltration. (B) Western blot analysis of agroinfiltrated leaves using anti-GFP antibody. Total proteins of infiltrating pGR107-GFP vector were used as a control.

Bax, as a pro-apoptotic member of the B cell lymphoma/leukemia (Bcl-2) family, triggers programmed cell death (PCD) (33). When fungal effectors are secreted into plant cells, they may exert its death-suppressive function. Therefore, we assayed the ability of *CcPEL*s to suppress Bax-induced PCD, which resembles the defense-related hypersensitive reaction (HR) in plant cells (34). In the tobacco transient expression assay, we overexpressed these seven *CcPELs* lacking their N-terminal SPs in Nicotiana tabacum leaves 24 h before infiltrating Bax into the agroinfiltration sites. Unexpectedly, the HR induced by infiltrating Bax into the each *CcPEL* transient overexpression area was not suppressed (Fig. 7A), even though Western blot analysis of agroinfiltrated leaves showed that these seven *CcPELs* were actually expressed (Fig. 7B). The results indicate that the seven *CcPELs* did not suppress Bax-induced cell death in N. tabacum.

### *CcPEL16* plays a role in the pathogenicity of *C. camelliae*.

**FIG 8:**
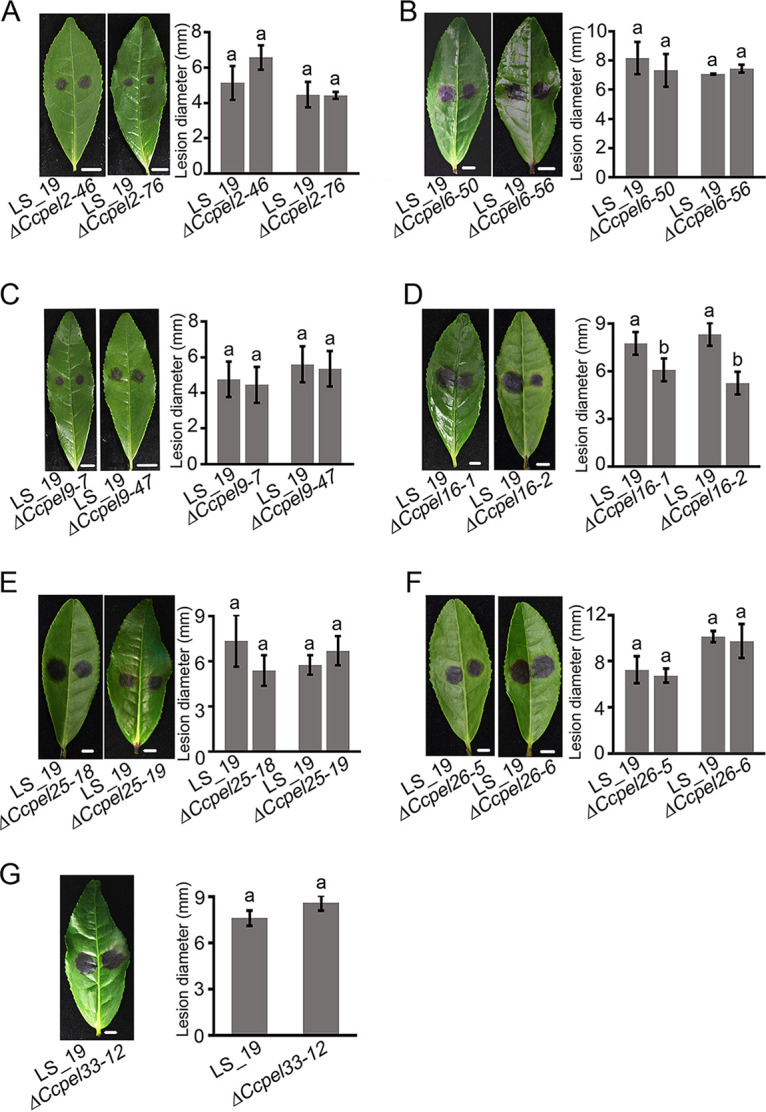
Pathogenicity assays of the *CsPEL* mutants in *C. camelliae*. Virulence of each strain was determined on detached tea leaves. Bar chart showing the lesion diameter caused by each tested strain. Lesions caused by the deletion mutants of *CcPEL2* (A), *CcPEL6* (B), *CcPEL9* (C), *CcPEL25* (E), *CcPEL26* (F), and *CcPEL33* (G) showed no significant differences compared with those caused by the wild-type strain LS_19. Necrotic lesions caused by the Δ*CcPEL16-1* and Δ*CcPEL16-2* mutants were significantly reduced (D). Same lowercase letters indicate no significant difference; different lowercase letters indicate significant differences (*P* < 0.05). Scale bar = 0.5 cm.

Although seven CcPEL proteins may not function as an attenuator of HR response in tobacco, the role of these *CcPELs* in virulence of *C. camelliae* toward the host *Ca. sinensis* needs to be further analyzed. We performed a pathogenicity analysis of *CcPEL* deletion mutants by inoculating *Longjing43* abraded leaves with conidial suspensions. By 4 dpi (days postinoculation), large necrotic lesions were observed at the inoculation sites of leaves inoculated with the wild-type strain LS_19, while much smaller lesions on inoculated leaves were seen for the Δ*CcPEL16-1* and Δ*CcPEL16-2* mutants (Fig. 8). However, there were no significant differences in lesion diameter between the other mutants and the LS_19 strain, indicating that the deletion of these genes did not affect the pathogenicity of *C. camelliae*. The results suggested that *CcPEL16* plays a role in the pathogenicity of *C. camelliae* on tea leaves.

## DISCUSSION

*C. camelliae* is one of the dominant pathogens causing foliar diseases in tea plants and can always be isolated from the diseased leaves (5, 35). In our previous studies, we have revealed the lifestyle characteristics and gene expression patterns of *C. camelliae* based on transcriptome data (3). As a hemibiotrophic pathogen, *C. camelliae* initially develops biotrophic hyphae inside host plant cells and then transitions to the necrotrophic stage (36). During infection, plant-colonizing fungi secrete effectors to directly dampen the plant immune system or redirect host processes, facilitating fungal growth (37). We have predicted many secreted proteins as potential candidate effectors that are significantly upregulated during *C. camelliae* infection of *Ca. sinensis*, including PCWDEs such as the carbohydrate esterases and PELs (3). These can induce the host innate immune defenses during infection, such as callose deposits and PCD (38). Although PELs have been shown to be involved in enhancing pathogenicity and inducing plant immune responses in several plant-pathogenic fungi, their biological functions as the candidate effectors in *C. camelliae* remain unknown.

Based on transcriptome data of *C. camelliae* LS_19, seven genes encoding PELs were identified in *C. camelliae*. qRT-PCR analyses further confirmed that *CcPEL* expression is induced and significantly upregulated during infection (Fig. 1), which suggested that the seven *CcPEL*s probably play important roles in the process of *C. camelliae* infection of tea plants. Gene deletion mutants of the *CcPEL* had to be generated to further analyze their molecular biological functions. Thus, we needed to establish an efficient genetic transformation system for *C. camelliae*. For filamentous fungi, two common methods used for genetic transformation are Agrobacterium tumefaciens-mediated transformation (ATMT) and PEG-mediated protoplast transformation (39). Compared with the complicated ATMT system, the PEG-mediated protoplast transformation system is particularly simple and efficient (40). It requires only a moderate amount of soluble wall-collapse enzymes, which has the advantages of simple operation, system maturation, and low instrument requirements. Therefore, it has been maturely applied in a variety of filamentous fungal pathogens, such as M. oryzae, Fusarium graminearum, and *Colletotrichum* spp. (41). In this study, we explored and optimized the PEG-mediated protoplast transformation system for *C. camelliae*. The optimum time for enzymatic hydrolysis was 7 h, with the young mycelia germinated from conidia under the proper osmotic buffer (Fig. 2C). The successful establishment of a PEG-mediated protoplast transformation system provides favorable conditions for the illumination of *CcPEL* biological functions in *C. camelliae*.

We generated targeted gene deletion mutants of seven *CcPEL*s, including *CcPEL2*, *CcPEL6*, *CcPEL9*, *CcPEL16*, *CcPEL25*, *CcPEL26*, and *CcPEL33*, respectively, via a homologous recombination strategy. Phenotypic analysis showed that all *CcPELs* except for *CcPEL33* are involved in vegetative growth (Fig. 3); *CcPEL2*, *CcPEL9*, *CcPEL25*, and *CcPEL26* are required for conidia production (Fig. 4); *CcPEL6*, *CcPEL25*, and *CcPEL26* are involved in regulating conidial morphology (Fig. 4C); and *CcPEL6*, *CcPEL16*, and *CcPEL26* are crucial for appressorium formation (Fig. 5). The results indicated that pectate lyases play important roles in the morphogenesis of *C. camelliae*. Surprisingly, few studies have reported the roles of PELs in the morphological development of pathogenic fungi. In *C. gloeosporioides*, the *in vitro* growth rate of *pelB* mutants showed no significant difference on glucose or Na polypectate media (22). In *Alternaria brassicicola*, colony sizes were similar for the Δ*pl1332-1* and wild-type strains on nutrient-rich PDA and minimal mineral agar supplemented with pectin, indicating that the pectate lyase-coding gene *PL1332* was dispensable for vegetative growth (42). In *C. magna*, two transformed isolates of *pel* were generated and selected for PEL activity and pathogenicity analyses, but lack of phenotypic analysis (43). However, the roles of *pelB* in *C. gloeosporioides*, *PL1332* in *A. brassicicola*, and *Cmpel* in *C. magna* in conidia production or appressorium development have not been studied. In this study, we first revealed the critical roles of *PEL* genes in vegetative growth, conidiogenesis, and appressorium development in *C. camelliae*. All seven CcPEL proteins in *C. camelliae* could be secreted into the plant cells (Fig. 6), suggesting their potential roles as effectors. A pathogenicity test on abraded *Ca. sinensis* leaves demonstrated that *CcPEL16* contributes to the virulence of *C. camelliae* (Fig. 8). Although PELs have been extensively reported to be involved in pathogenesis in phytopathogenic fungi (19, 21–26), the role of *PEL16* in pathogenicity is rarely reported. In *P. capsici*, the pectate lyase gene *PcPL16* showed strong expression levels during infection, and overexpressing *PcPL16* triggered strong cell death in pepper and tobacco leaves (25). The silenced line 16-S11 (*PcPL16*) produced significantly smaller lesions on pepper leaves compared with the aggressive isolate SD33, suggesting that *PcPL16* makes an important contribution to virulence (25). In this study, we first demonstrated that *CcPEL16* plays a critical role in the virulence of *C. camelliae* against tea plants.

PELs can manipulate host immune responses as the pathogen-associated molecular patterns (PAMPs) or damage-associated molecular patterns (DAMPs) (44). For example, VdPEL1 in *V. dahlia* was secreted into plant cells to degrade pectin in the roots, resulting in the release of the plant cell wall fragments (DAMPs) and triggering defense responses (19). Here, cell death-suppressive activity assays showed that CcPEL16 did not suppress Bax-induced cell death in N. tabacum leaves (Fig. 7), indicating that it did not trigger the plant defense responses in tobacco. However, many studies have shown that functional effectors contributing to pathogen virulence strongly induce plant cell death in tobacco leaves, such as F. graminearum Fg12, Phytophthora sojae PsCRN63, and *P. sojae* Avh241 (45–47). Therefore, we further performed pathogenicity analyses in host tea leaves to determine the role of CcPELs in *C. camelliae* virulence. When susceptible tea leaves were inoculated with conidial suspensions, the Δ*Ccpel16* mutants caused significantly reduced necrotic lesions compared to the wild-type strain LS_19 (Fig. 8). The result indicates that *CcPEL16* as a virulence factor is essential for the pathogenicity of *C. camelliae* against tea plants. The attenuated virulence of the Δ*CcPEL16* mutants may be due to the significant decrease in appressoria as the infection structure. Considering the induced high expression during infection (Fig. 1) and the presence of a functional secretory SP (Fig. 6), we speculated that CcPEL16 in *C. camelliae* may act as an effector or PAMP to elicit immune defenses in its own host *Ca. sinensis*. In subsequent studies, we need to confirm whether tea plants can recognize PEL degradation products which do or do not act as DAMPs. In addition, the PAMP activity of CcPEL16 protein needs to be further confirmed in the future.

### Conclusions.

In this study, we found that seven *CcPEL* genes in *C. camelliae* were significantly upregulated expressed during infection. To further confirm their biological functions, we generated targeted gene deletion mutants of seven *CcPELs* via a homologous recombination strategy with an efficient PEG-mediated protoplast transformation system in *C. camelliae*. Phenotypic analysis revealed that pectate lyase genes are involved in vegetative growth, conidiogenesis, and appressorium development in *C. camelliae*. All seven CcPEL proteins contained SPs at the N terminus and could be secreted. Furthermore, deletion of *CcPEL16* impaired the virulence of *C. camelliae* against *Ca. sinensis*. However, the roles of CcPEL16 protein as a candidate effector needs to be further confirmed in the future.

## MATERIALS AND METHODS

### Fungal strains and culture conditions.

The wild-type *C. camelliae* strain LS_19 was previously isolated from tea plants in China (27). Strain LS_19 and the *CcPEL* mutants were cultured on PDA (20% potato, 2% d-glucose, and 1.5% agar) medium at 28°C in the dark.

### RNA isolation and quantitative RT-PCR analyses.

Total RNA was extracted from tea leaves at 3, 6, 24, 36, 48, 72, and 96 h after inoculation with LS_19 using FastPure Universal Plant Total RNA Isolation kit (Nanjing Vazyme Biotech Co., Ltd., China), and then the first strand of cDNA was synthesized with reverse transcription by HiScript II Q RT SuperMix for qPCR (+gDNA wiper) (Nanjing Vazyme Biotech Co., Ltd., China). qRT-PCR analysis was performed with ChamQ SYBR qPCR Master Mix (Nanjing Vazyme Biotech Co., Ltd., China) using the Bio-Rad CFX96 Real-Time System. The primers used to amplify the seven *CcPEL*s and *CcNEW1* used for qRT-PCR assays are listed in Table S1. The relative quantification of each transcript was calculated as previously described (28, 48). All qRT-PCR analyses were conducted in three replicates for each sample and three biological replicates were maintained.

### Generation of *CcPEL* deletion mutants.

All *CcPEL* sequences were obtained from the genome data (GenBank: JAELVL010000001.1) of *C. camelliae*. The primer pairs used to construct the *CcPEL* gene replacement vectors, the *HPH* gene cassette, and the upstream and downstream flanking fragments are listed in Table S1. The fused fragments of each *CcPEL* gene were cloned into the vector pKOV21 using the CloneExpress Multis reaction system (Nanjing Vazyme Biotech Co., Ltd., China) (49). The gene replacement vectors were then transformed into protoplasts of the wild-type strain LS_19 to generate null mutants. All gene deletion mutants were confirmed by PCR assays.

### Phenotypic analysis.

For assessment of the colony growth of each strain, 5-mm diameter mycelial plugs cut from the edge of a 5-day-old colony were placed on PDA plates and incubated at 28°C for 4 days. Conidiation assays of all strains were performed after culturing for 7 days on PDA medium. The conidia produced on PDA plates were scraped off and then filtrated. Next, conidia concentrations were measured using a hemocytometer. The conidial size of each strain was determined using ImageView software under light microscopy. At least 50 conidia were measured. For appressorium development analysis, conidial suspensions with a concentration of 1 × 10^5^ conidia/mL were cultured on hydrophobic coverslips in the dark. The appressoria of each strain were examined under a light microscope at 24 hpi. All experiments were repeated three times with at least three replicates.

### SP activity assays.

The predicted SPs of *CcPEL* were fused with the vector pSUC2. Constructed recombinant vectors were transformed into the yeast strain YTK12 as described previously (32). All transformants were cultured on CMD-W medium (6.7 g/L yeast nitrogen base without amino acids, 0.75 g/L tryptophan dropout supplement, 20 g/L agar, sterilization at 121°C for 20 min, addition of 2% sucrose, 0.1% d-glucose) to select positive colonies (8). To detect invertase secretion, the positive transformants were cultured on YPRAA medium (10 g/L yeast extract, 20 g/L peptone, 20 g/L raffinose, 20 g/L agar, sterilization at 121°C for 20 min, addition of 20 μg/mL antimycin A). For the color reaction, the positive transformants were suspended with the suspension buffer (1.5 mM acetic acid-sodium buffer [pH 4.7], 3.5% sucrose solution). After centrifugation of the suspension, the supernatant was added to 0.1% TTC to detect the color and then photographed.

### Cell-death inhibition assay.

Each *CcPEL* cDNA sequence (without SP sequence) was cloned into the vector pGR107-GFP. Each constructed vector, respectively, was transformed into A. tumefaciens GV3101. For transient overexpression of *CcPEL*s in N. tabacum, recombinant strains of A. tumefaciens were cultured in LB liquid medium with 50 μg/mL kanamycin and 50 μg/mL rifampicin at 28°C for 24 h. Cells were resuspended and adjusted to an optical density at 600 nm (OD_600_) of 0.5, and then injected into the leaves of 5-week old N. tabacum plants. A. tumefaciens cells harboring the *BAX* gene were inoculated into the same sites on the tobacco leaves at 24 hpi. The same leaves were injected with A. tumefaciens carrying an empty vector pGR107-GFP as a control. Cell death symptoms were monitored at 7 dpi and photographed. All tests were performed with three replicates.

### Western blot analysis.

Total proteins were extracted from the leaf infiltrated area as previously described (47). Protein samples were fractioned by 10% SDS-PAGE gel and electroblotted onto polyvinylidene difluoride (PVDF) membrane, then immunoblotted with anti-GFP antibody (Abmart, China) at a dilution of 1:5,000. HiSec horseradish peroxidase-conjugated goat anti-mouse IgG (H+L) (Nanjing Vazyme Biotech Co., Ltd., China) was used as the secondary antibody. The chemiluminescence signals were detected with a FDbio-Femto RCL kit (FDbio Science, China).

### Pathogenicity assays.

Healthy and non-wounded mature leaves collected from a 5-year-old *Ca. sinensis* cv. *Longjing* 43 grown in a tea garden in Hangzhou, Zhejiang Province, China, were washed with sterilized water and then inoculated using wound inoculation methods (35, 50). Conidial suspensions of the wild-type strain LS_19 at a concentration of 1 × 10^5^ conidia/mL were inoculated on the left sides of leaves, and conidial suspensions of the *CcPEL* mutants at the same concentration were inoculated on the right sides. Symptomatic lesions and lesion diameters were determined at 4 dpi. Experiments were repeated three times with three replicates for each strain.

### Light and fluorescence microscopy.

Light microscopy was used to visualize protoplasts, conidia, and appressoria of *C. camelliae*. Fluorescence microscopy was used to visualize GFP. The exciation and emission wavelengths used were 488 nm and 507 nm.
