# Supplementary figures and images for: Pectate Lyase Genes Abundantly Expressed During the Infection Regulate Morphological Development of Colletotrichum camelliae and CcPEL16 Is Required for Full Virulence to Tea Plants
Source: mSphere. 2023 Jan 24;8(1):e00677-22. doi: 10.1128/msphere.00677-22 (PMC9942558; doi:10.1128/msphere.00677-22)

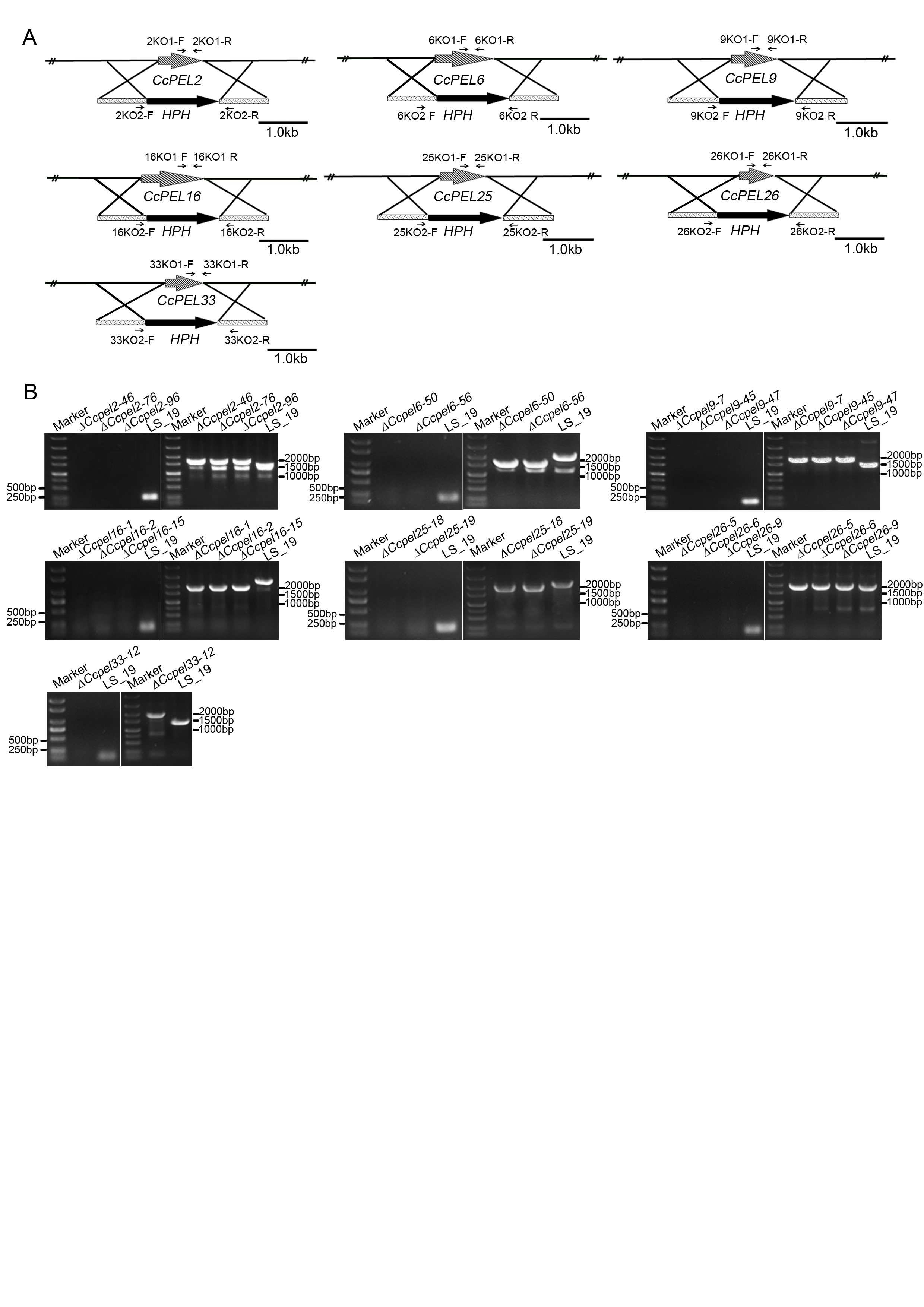

Supplement: FIG S1 [file msphere.00677-22-s0002.jpg]
