# Supplementary material for: Pectate Lyase Genes Abundantly Expressed During the Infection Regulate Morphological Development of Colletotrichum camelliae and CcPEL16 Is Required for Full Virulence to Tea Plants
Source: mSphere. 2023 Jan 24;8(1):e00677-22. doi: 10.1128/msphere.00677-22 (PMC9942558; doi:10.1128/msphere.00677-22)
Supplement: TABLE S1 [file msphere.00677-22-s0001.docx]

**Table S1.** PCR primers used in this study.

| Primer | Sequence (5’-3’) | Relevant characteristics |
| --- | --- | --- |
| 2 up-F | CCCCCGGGCTGCAGGAATTCATCCGCCCAGATAGCAAA | Amplification of upstream fragment of *CcPEL2* for the construction of deletion mutants |
| 2 up-R | GCTCCTTCAATATCATCTTCTCTCGACAGTTCCCGACCATAAA |  |
| 2 down-F | TAGAGTAGATGCCGACCGAACAAGACCAGCACAGTCAAACCCG | Amplification of downstream fragment of *CcPEL2* for the construction of deletion mutants |
| 2 down-R | TACCGGGCCCCCCCTCGAGGATTCCAGTGGCAGAGG |  |
| 2YW-F | CAGTGTAAGCGGAATGTG | For identification of *CcPEL2* gene deletion transformants (presence or not), or qRT-PCR analysis of *CcPEL2* expression |
| 2YW-F | CCAGAGCAAACGCCAGAC |  |
| 2DX-F | TTCTCACAGTTGCGTTCC | For identification of *CcPEL2* gene deletion transformants (size difference) |
| 2DX-R | GTAAGGGCGATCATCTTG |  |
| 6 up-F | CCCCCGGGCTGCAGGAATTCTTTCCCGTCATCAATCGC | Amplification of upstream fragment of *CcPEL6* for the construction of deletion mutants |
| 6 up-R | GCTCCTTCAATATCATCTTCTCTCGCTGGGCAGCAGCTCACTT |  |
| 6 down-F | TAGAGTAGATGCCGACCGAACAAGAGACCAATGCTGGCTACCT | Amplification of downstream fragment of *CcPEL6* for the construction of deletion mutants |
| 6 down-R | TACCGGGCCCCCCCTCGAGCGCAATCCTGGAGTTCCT |  |
| 6YW-F | CTTGGTTACATCTGCGAGTG | For identification of *CcPEL6* gene deletion transformants (presence or not), or qRT-PCR analysis of *CcPEL6* expression |
| 6YW-R | TTGGTGCTAGTAGTCCTTCC |  |
| 6DX-F | AAACCGTTGCATATTTGGG | For identification of *CcPEL6* gene deletion transformants (size difference) |
| 6DX-R | GCCCGGATAACTAGACGT |  |
| 9 up-F | CCCCCGGGCTGCAGGAATTCCCCCTGACTTCATTTCGC | Amplification of upstream fragment of *CcPEL9* for the construction of deletion mutants |
| 9 up-R | GCTCCTTCAATATCATCTTCTCTCGTTTGGCTGGTTTGTGGTAG |  |
| 9 down-F | TAGAGTAGATGCCGACCGAACAAGAAGTGGGTAATGAGAAGAGCG | Amplification of downstream fragment of *CcPEL9* for the construction of deletion mutants |
| 9 down-R | TACCGGGCCCCCCCTCGAGAATGCCTTGGATGGTGGT |  |
| 9YW-F | CAACAACTACGCCGAGAAGC | For identification of *CcPEL9* gene deletion transformants (presence or not), or qRT-PCR analysis of *CcPEL9* expression |
| 9YW-R | TGTAGTCGGGCTTGGTGAA |  |
| 9DX-F | ATCCCGCTTCACTTCTTC | For identification of *CcPEL9* gene deletion transformants (size difference) |
| 9DX-R | ATTCACAGAACCAGGAGC |  |
| 16 up-F | CCCCCGGGCTGCAGGAATTCAAAGATGTATCCGTAGGTCAGGTTG | Amplification of upstream fragment of *CcPEL16* for the construction of deletion mutants |
| 16 up-R | GCTCCTTCAATATCATCTTCTCTCGGGGAAAGAATCGAAACAAAGAGTG |  |
| 16 down-F | TAGAGTAGATGCCGACCGAACAAGATGGTTCCCACAATGAGGCTGAC | Amplification of downstream fragment of *CcPEL16* for the construction of deletion mutants |
| 16 down-R | TACCGGGCCCCCCCTCGAGACGAACTCCCAGCCGACCTG |  |
| 16YW-F | GCCGCCCTCGTCAAGCAGAT | For identification of *CcPEL16* gene deletion transformants (presence or not), or qRT-PCR analysis of *CcPEL16* expression |
| 16YW-R | CGTTCAACCCGTTGGCAGTC |  |
| 16DX-F | ACCCACGGTTCTTCAGGT | For identification of *CcPEL16* gene deletion transformants (size difference) |
| 16DX-R | TGTTGGATGCGGATTTCA |  |
| 25 up-F | CCCCCGGGCTGCAGGAATTCGGCATTGAGTGGAGGAGTGT | Amplification of upstream fragment of *CcPEL25* for the construction of deletion mutants |
| 25 up-R | GCTCCTTCAATATCATCTTCTCTCG GGTCAAAGAAGCCGCAAAA |  |
| 25 down-F | TAGAGTAGATGCCGACCGAACAAGAGAGCCTTTCTCCTTGATCTCCC | Amplification of downstream fragment of *CcPEL25* for the construction of deletion mutants |
| 25 down-R | TACCGGGCCCCCCCTCGAGGGTCGCTTGTGGTCCATCTAA |  |
| 25YW-F | ATGGGCATCGTAAGTGTT | For identification of *CcPEL25* gene deletion transformants (presence or not), or qRT-PCR analysis of *CcPEL25* expression |
| 25YW-R | GTAGGGTTCTTCGGGTTC |  |
| 25DX-F | TTGATTCACGGAAACTCG | For identification of *CcPEL25* gene deletion transformants (size difference) |
| 25DX-R | ACCTTGTCCGACGTGTCT |  |
| 26 up-F | CCCCCGGGCTGCAGGAATTCGACGCCGTAGGTGACATTAGC | Amplification of upstream fragment of *CcPEL26* for the construction of deletion mutants |
| 26 up-R | GCTCCTTCAATATCATCTTCTCTCGGTCTGCCATCTTCCAGGATTT |  |
| 26 down-F | TAGAGTAGATGCCGACCGAACAAGATCGGGCTCAATCCTCTGG | Amplification of downstream fragment of *CcPEL26* for the construction of deletion mutants |
| 26 down-R | TACCGGGCCCCCCCTCGAGGACTCCCTTTCTTCTGTCGTTTT |  |
| 26YW-F | GCGGAGGACTACGGCAAAG | For identification of *CcPEL26* gene deletion transformants (presence or not), or qRT-PCR analysis of *CcPEL26* expression |
| 26YW-R | GCACTCGCCTGACTTGG |  |
| 26DX-F | TTGATTGTGCAGCGTGAT | For identification of *CcPEL26* gene deletion transformants (size difference) |
| 26DX-R | GACGCACAGAATATGGAGTT |  |
| 33 up-F | CCCCCGGGCTGCAGGAATTCGCGTATCCTCTTAACCTTTG | Amplification of upstream fragment of *CcPEL33* for the construction of deletion mutants |
| 33 up-R | GCTCCTTCAATATCATCTTCTCTCGCAACGAATGAATAACCACC |  |
| 33 down-F | TAGAGTAGATGCCGACCGAACAAGACTTCGCTCGGTCTTCGT | Amplification of downstream fragment of *CcPEL33* for the construction of deletion mutants |
| 33 down-R | TACCGGGCCCCCCCTCGAGGGCGGGATTGCTCTTGGA |  |
| 33YW-F | CGGCATCAACTCCAACAAG | For identification of *CcPEL33* gene deletion transformants (presence or not), or qRT-PCR analysis of *CcPEL33* expression |
| 33YW-R | GCCGGAGCCGATCTTCTT |  |
| 33DX-F | GCTGAGGGATAGGACTGC | For identification of *CcPEL33* gene deletion transformants (size difference) |
| 33DX-R | CATCATGGCAAATTGGTT |  |
| PEL2-BAX-F | GTCAGCACCAGCTAGCAATGTGTCTCGGCTACGA | Amplification of full length ORF of *CcPEL2* (without SP sequence) for the cell-death inhibition assay |
| PEL2-BAX-R | TCAAGCTTATCGGCGGTCGACTTATCCAGAGCAAACGC |  |
| PEL6-BAX-F | GAGGTCAGCACCAGCTAGCAATGAAGGTCTCGTTCC | Amplification of full length ORF of *CcPEL6* (without SP sequence) for the cell-death inhibition assay |
| PEL6-BAX-R | TCAAGCTTATCGGCGGTCGACCTAAGAGTTGGTGCTAGTAG |  |
| PEL9-BAX-F | GTCAGCACCAGCTAGCAATGACCCCCACCCCTACCGT | Amplification of full length ORF of *CcPEL9* (without SP sequence) for the cell-death inhibition assay |
| PEL9-BAX-R | TCAAGCTTATCGGCGGTCGACTTAGAAGGTGAGGGTCTGGC |  |
| PEL16-BAX-F | GAGGTCAGCACCAGCTAGCAATGAAGGCTTCCATCTCTCT | Amplification of full length ORF of *CcPEL16* (without SP sequence) for the cell-death inhibition assay |
| PEL16-BAX-R | TCAAGCTTATCGGCGGTCGACTCAGTAAACAGCCGGGG |  |
| PEL25-BAX-F | GAGGTCAGCACCAGCTAGCAATGAAGATCGCCTCTACCT | Amplification of full length ORF of *CcPEL25* (without SP sequence) for the cell-death inhibition assay |
| PEL25-BAX-R | TCAAGCTTATCGGCGGTCGACCTAGTTGAAGCCCTGGGG |  |
| PEL26-BAX-F | GAGGTCAGCACCAGCTAGCAATGCGCACCGACGCCTTCAA | Amplification of full length ORF of *CcPEL26* (without SP sequence) for the cell-death inhibition assay |
| PEL26-BAX-R | TCAAGCTTATCGGCGGTCGACCTAGCACTCGCCTGACTT |  |
| PEL33-BAX-F | GAGGTCAGCACCAGCTAGCAGCCCCCGGTGGCCAGTCTG | Amplification of full length ORF of *CcPEL33* (without SP sequence) for the cell-death inhibition assay |
| PEL33-BAX-R | TCAAGCTTATCGGCGGTCGACTTAGCAAGCCTTGAGGGTG |  |
| HPH-F | TATTGAAGGAGCATTTTTGG | Amplification of *HPH* gene cassette |
| HPH-R | GCTCTTGTTCGGTCGGCATC |  |
| Ccnew1-F | CGTGGCTTTGAAATCAGACC | qRT-PCR analysis of *CcNEW1* expression |
| Ccnew1-R | CCATCTCGTGACTAGGAGCAA |  |
